# Beyond a unitary Simon effect: dissociating stimulus- and response-driven spatial interference across finger and leg responses

**DOI:** 10.1007/s00426-026-02354-x

**Published:** 2026-07-30

**Authors:** Fereidoun Malaei, Jamin Halberstadt

**Affiliations:** Department of Psychology, University of Otago, PO Box 56, 9054 Dunedin, New Zealand

**Keywords:** Cognitive control, Conflict adaptation, Response effector, Simon effect, Sensorimotor asymmetry

## Abstract

The Simon effect—faster and more accurate responding when stimulus location corresponds to response side—reflects competition between goal-directed response selection and automatic spatial coding. Although typically treated as a single performance index, this aggregate measure obscures the distinct contributions of stimulus location (left vs. right) and response side (left vs. right) to spatial interference. The present study examined how effector type shapes stimulus- and response-related spatial interference by comparing seated finger presses and standing leg taps in the Simon task (*N* = 32). Treating stimulus location and response side as independent analytical factors revealed four stimulus- and response-driven interference contrasts for each effector. For reaction times, all four contrasts were significant for leg responses, whereas three were significant for fingers, with the right response-driven contrast the largest (58.9 ms) and the left response-driven the smallest (27.1 ms). For errors, significant effects were observed for fingers only, in the right response-driven (6.80%) and left stimulus-driven (6.49%) contrasts. The congruency sequence effect—reduced interference following incongruent relative to congruent trials—was significant for both effectors in reaction times, with a larger numerical reduction for fingers (77.2%) than legs (43.9%), but significant only for fingers in errors, where interference was eliminated and marginally reversed following incongruent trials. The four interference contrasts decreased substantially across blocks for leg responses — both in absolute and proportional terms — but remained largely stable for fingers. These findings highlight effector-specific patterns of conflict resolution, with potential implications for cognitive–motor assessment and rehabilitation.

## Introduction

In principle, a system that responds exclusively to task-relevant information should ignore irrelevant stimulus features. Consider a simple robot programmed to respond only to stimulus colour: a red circle triggers a left-button press, whereas a green circle triggers a right-button press. Because the correct action is determined solely by colour, performance would be unaffected by stimulus location, with reaction times (RTs) and accuracy remaining equivalent across positions, as spatial position is task-irrelevant and carries no information about the correct response. However, when humans perform the same task, responses are consistently faster and more accurate when the spatial location of a stimulus corresponds to the required response side, even though location remains task-irrelevant. This systematic advantage for spatially corresponding stimulus–response pairings is known as the Simon effect (Hommel, 2011; Simon, 1990) and has been attributed to competition between goal-directed response selection and automatic activation of spatially corresponding response codes (for review, see Cespón et al., 2020).

The Simon effect arises not only from the parallel activation of automatic and controlled response pathways (Kornblum et al., 1990; Proctor & Vu, 2006) but also from trial history (Botvinick et al., 2001; Hommel et al., 2004; Stürmer et al., 2002). As documented across inhibitory control paradigms (Egner & Hirsch, 2005; Gratton et al., 1992; Lamers & Roelofs, 2011), the spatial interference typically diminishes following incongruent trials—a phenomenon referred to as the congruency sequence effect (CSE, Cespón et al., 2020; Gratton et al., 1992). This sequential modulation is consistent with dynamic upregulation of cognitive control, whereby conflict on a preceding trial enhances top-down attention and response selection on the subsequent trial (Botvinick et al., 2001; Stürmer et al., 2002), although feature repetition and event-file binding mechanisms may also contribute (Hommel et al., 2004; Mayr et al., 2003). Despite extensive replication of the Simon effect across motor modalities, including finger and leg responses (Chen et al., 2021; Proctor et al., 2023), it remains unclear whether the CSVis similarly modulated by effector type.

Traditionally, the Simon effect has been treated as a unitary performance measure (Cespón et al., 2020). However, this aggregation obscures the dissociable contributions of stimulus location and response side. Left- and right-sided stimuli engage distinct hemispheric attentional systems, whereas left- and right-sided responses differ in corticospinal recruitment and motor lateralisation (Proctor et al., 2023; Seibold et al., 2016; Tagliabue et al., 2007). As shown by Tagliabue et al. (2007), congruency-based analyses mathematically conflate these influences, such that apparent asymmetries in the Simon effect may instead reflect response-side differences in dominant versus non-dominant effector speed rather than genuine spatial compatibility effects. Separating stimulus position and response side into independent analytical factors therefore permits a more precise characterisation of whether spatial interference arises primarily from stimulus-driven attentional processing, response-related motor execution, or their interaction — revealing lateralised asymmetries and effector-specific biases that remain concealed within a unitary congruency index. This distinction is important for advancing theoretical accounts of cognitive control and for clinical applications requiring greater measurement precision.

Given that distinct neural systems underlie different effector types and goal-directed actions, cognitive performance may be shaped by the type of motor engagement (Dum et al., 2016; Gordon et al., 2023; Graziano, 2016). For instance, posture—such as sitting versus standing—has been shown to modulate performance in the Stroop task (Abou Khalil et al., 2024). In addition, evidence suggests that using the non-dominant hand in problem solving may recruit broader neural resources, such as visuomotor networks, potentially enhancing learning outcomes (Coudiere et al., 2024; Kirby et al., 2019; Park & Son, 2022). These findings highlight essential aspects of human adaptive cognitive-motor capacities, including sensorimotor processes, motor lateralization, and the distinct neurocognitive demands of dominant vs. non-dominant response use (Serrien & Sovijärvi-Spapé, 2013; Walsh et al., 2008). Considering these adaptive capacities in spatial conflict resolution can advance current top-down theoretical models of the Simon effect (Cespón et al., 2020) and inform practical strategies to enhance cognitive control.

Moreover, resolving spatial conflict requires the flexible recruitment of prefrontal–motor networks to override automatic spatial codes and prioritize task-relevant responses (Friedman & Robbins, 2022). Given the adaptive capacity of these neural pathways to reorganize with experience, repeated task exposure is expected to attenuate spatial interference (Proctor & Lu, 1999). Such practice-related modulations may vary depending on the effector type, reflecting the close interplay between cognitive control mechanisms and sensorimotor dynamics (Gentili et al., 2015; Koziol et al., 2012; Sherman & Usrey, 2021; Volz et al., 2015). However, the extent to which left- and right-effector involvement modulates the Simon effect across repeated task blocks remains poorly understood.

Finally, unlike a robot executing programmed rules without neurobiological adaptation, human cognition evolved in close interaction with ecological demands — navigation, foraging, and real-time engagement with a dynamic environment. Meeting these demands required upright posture and coordinated lower-limb movement, shaping tightly integrated cognitive–motor systems (Bramble & Lieberman, 2004; Malaei et al., 2022; Malaei, 2025; Raichlen et al., 2017). Yet modern cognitive control research relies predominantly on seated participants and manual responses, thereby removing cognition from the embodied contexts in which it evolved (Siakaluk et al., 2011; Wilson, 2002). Consequently, relatively little is known about how cognitive control operates during standing lower-limb actions that more closely resemble natural human behaviour (Chen et al., 2021). The present research addresses this gap by examining the Simon effect using both seated finger-press responses and standing leg taps, testing whether spatial interference and its sequential modulation generalise across effector systems that differ in their embodied and evolutionary constraints.

## Aims and hypotheses

The present study examined how variations in motor systems influence performance in the Simon task. To extend the conventional framework of the Simon effect, we compared seated left- and right-hand finger responses with standing left- and right-leg responses, using an analytical approach that treats stimulus location and response side as independent factors (Proctor et al., 2023; Seibold et al., 2016; Tagliabue et al., 2007). This approach allows the Simon effect to be decomposed into stimulus-driven and response-driven components, providing a more fine-grained characterization of spatial interference. We additionally tested whether CSE differ across response modalities. We predicted that the Simon effects and its sequential modulations would be present in both effectors but would differ in magnitude. Specifically, we hypothesized that finger responses would show more efficient conflict resolution, reflected in a smaller Simon effect and stronger sequential adaptation. In contrast, we expected leg responses to exhibit a larger initial Simon effect but greater attenuation of interference across experimental blocks, consistent with adaptive recruitment of less frequently used motor systems. Together, these predictions aim to clarify how cognitive control interacts with effector-specific motor dynamics in resolving spatial conflict.

## Method

### Participants

Thirty-two right-handed undergraduate students (6 males, 26 females, aged 18–33 years, M = 20.13, SD = 3.17) from the Department of Psychology participant pool at the University of Otago participated in the study in exchange for course credit. All participants reported normal or corrected-to-normal vision and no history of neurological or motor impairments. Their average height and weight were 168 cm (SD = 8.10) and 66 kg (SD = 10.92). Ethical approval was obtained from the University Ethics Committee, and all participants provided written informed consent before the experiment.

### Materials, stimuli, and procedure

The Simon task was implemented using the Psychology Experiment Building Language (PEBL; Mueller & Piper, 2014), which controlled stimulus presentation and response recording. Visual stimuli, consisting of colored circles (each with a radius of 50 pixels), were presented on a 23.8-inch Dell P2422H monitor (1920 × 1080 resolution, 60 Hz IPS panel) against a black background. The monitor was wall-mounted with an adjustable height to ensure a consistent viewing angle across both effector conditions (finger and leg) and participants of varying heights. Participants viewed the monitor from 60 cm away, with the circles appearing 11.5° visual angle to the left or right of a central fixation cross. Four colors (red, green, purple, and yellow) were used for stimuli, with two specifically assigned to the finger effector and the remaining two to the leg effector. The color-to-response mapping was counterbalanced across participants. The target stimulus remained on the screen until a response was made. Response times (RT), measured in milliseconds (ms), were recorded from the stimulus onset to the participant’s button press.

**Fig. 1:**
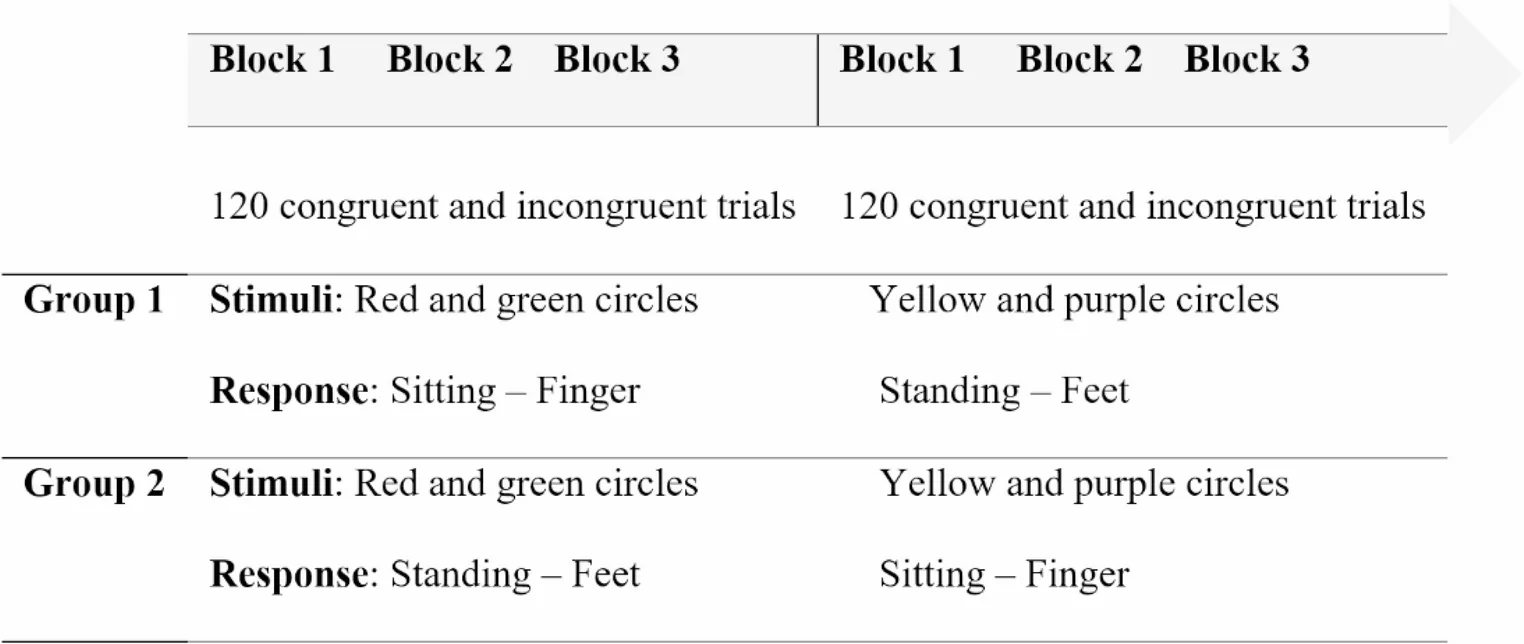
Procedure. Participants completed six Simon task blocks (three finger, three leg), counterbalanced across participants

Each participant completed the Simon task under both finger (i.e., index finger presses) and leg (i.e., foot taps) conditions, with the order counterbalanced across participants (see Fig. 1). In both conditions, participants completed 12 practice trials with feedback, followed by 120 experimental trials (congruent and incongruent) and 20 neutral trials (stimuli presented at the centre). Within the experimental trials, each of the four stimulus–response combinations (left–left, left–right, right–left, right–right) was presented 30 times per effector condition, yielding 30 trials per cell prior to exclusions — within the range considered sufficient for stable within-subject RT estimates (Ratcliff, 1993; Whelan, 2008).

Each effector condition comprised three blocks, separated by two short breaks lasting approximately 5–7 s. Within each block, stimulus colors and spatial positions were pseudo-randomized — using a fully counterbalanced and shuffled design — to ensure equal numbers of congruent and incongruent trials, while minimizing sequence predictability. The pseudo-randomisation produced approximately equal numbers of congruent–congruent, congruent–incongruent, incongruent–congruent, and incongruent–incongruent trial transitions within each block, as an equal distribution of congruent and incongruent trials across the sequence necessarily yields approximately balanced transition frequencies. Transition probabilities were not, however, explicitly constrained beyond this equal-frequency requirement on current-trial congruency, and residual imbalances cannot be ruled out.

In the finger condition, participants were seated at a desk and responded using their left and right index fingers to press response buttons (30 cm × 30 cm) positioned beneath the tabletop. In the leg condition, participants stood upright and responded by tapping the same buttons, now placed on the floor, with their left or right foot. In both conditions, participants viewed only the screen and did not have visual access to the response buttons. In both conditions, participants were instructed to respond as quickly and accurately as possible by pressing the left key for one color (e.g., red) and the right key for another (e.g., blue), while ignoring the circle’s position on the screen. Stimuli, timing parameters, and task instructions were kept consistent across response modalities.

### Design

Following the data analysis approach used in previous studies (Chen et al., 2021; Proctor et al., 2023; Seibold et al., 2016; Tagliabue et al., 2007), our experiment employed a fully within-subjects design with three independent variables: stimulus position (left vs. right), response side (left vs. right), and effector type (finger vs. leg). The dependent variables were RT and percentage of errors (PE).

### Data analysis

Mean RTs and error rates were analyzed using repeated-measures ANOVAs to assess the effects of effector type on the Simon effect, CSV, and practice-related changes. Previous research has adopted diverse approaches to outlier exclusion in RT data preparation (for a review, see Miller, 2023). Only correct congruent and incongruent trials preceded by correct responses were retained. Neutral trials were excluded as they lack a defined congruency relation and were included only to minimise strategic anticipation of stimulus location. Outliers were removed in two steps: first via a broad fixed cutoff, then via an interquartile range (IQR) filter to exclude values deviating substantially from each participant’s typical performance.

**Fig. 2:**
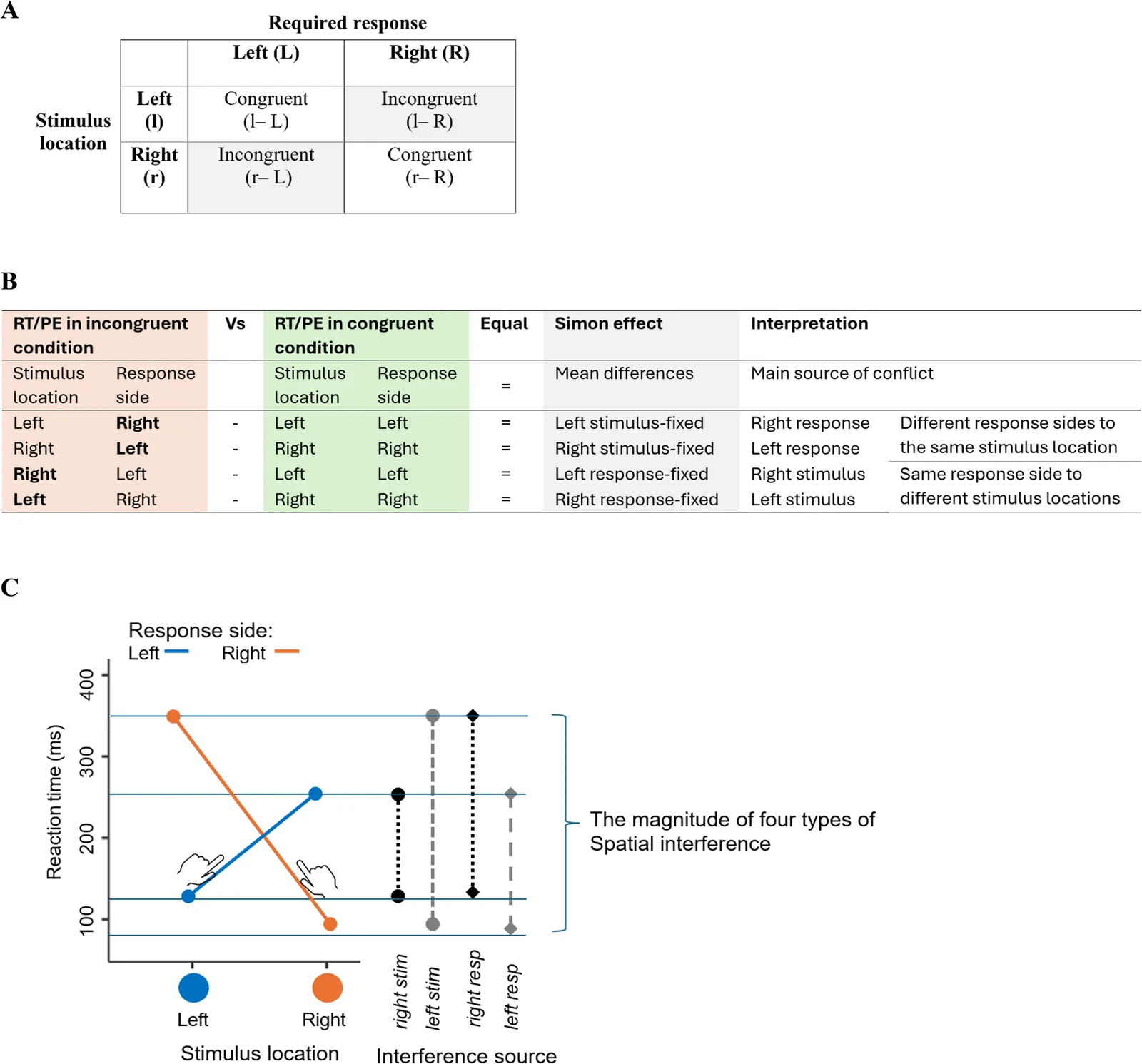
Four interference contrasts in the Stimulus × Response space. Panel **A** shows the stimulus–response congruency matrix, in which the four combinations of stimulus location (left, right) and required response (left, right) are classified as congruent or incongruent. Panel **B **shows the decomposition of this matrix into four pairwise contrasts between incongruent (left–right, right–left) and congruent (left–left, right–right) conditions, isolating spatially distinct sources of interference across stimulus–response configurations. Panel **C **visualizes the four spatial interference contrasts, showing schematic mean RT differences between incongruent and congruent conditions. Each contrast distinguishes response-driven interference, in which stimulus location is held constant, from stimulus-driven interference, in which response side is held constant. Values are hypothetical and for illustrative purposes only

For RT, a 2 × 2 × 2 repeated-measures ANOVA with stimulus position (left, right), response side (left, right), and effector (finger, leg) as within-subjects factors was conducted. The stimulus position × response side interaction captured the overall Simon effect from both response-driven and stimulus-driven perspectives (see Fig. 2), with effector added to test whether the effect differed across effectors. The CSVwas examined via the three-way interaction of stimulus position × response side × previous congruency, and the four-way interaction tested whether conflict adaptation differed across effectors.

Practice effects were analysed using a 2 × 2 × 2 × 2 design with stimulus position, response side, effector, and block (Block 1 vs. Block 3) as within-subjects factors. Block 2 was excluded as a transitional phase, reducing model complexity and mitigating potential sphericity violations. Identical analyses were conducted for error rates. Significant effects were followed by Bonferroni-corrected pairwise comparisons (see Fig. 2B), and both ηp² and ηG² are reported to allow comparison across within-subject designs (Bakeman, 2005; Olejnik & Algina, 2003).

## Results

### RT Simon effect

Trials with errors (2.9%) and extreme RTs (< 200 ms or > 2000 ms for fingers; <300 ms or > 2500 ms for legs) were excluded (0.2%). An IQR filter across the eight conditions removed 4.8% of trials. In total, 7.9% of experimental trials were excluded. As described above, a 2 × 2 × 2 × 2 repeated-measures ANOVA was conducted on RTs to examine the effects of stimulus position, response side, previous-trial congruency, and effector on RT.

The main effects of stimulus location, *F*(1, 31) = 0.01, *p* = .909, ηp² < 0.001 (ηG² ≈ 0.000), response side, *F*(1, 31) = 1.01, *p* = .322, ηp² = 0.032 (ηG² = 0.001), and previous trial, *F*(1, 31) = 0.18, *p* = .678, ηp² = 0.006 (ηG² ≈ 0.000) were not significant. There was a significant main effect of effector, F(1, 31) = 918.37, *p* < .001, ηp² = 0.967 (ηG² = 0.848), with RTs being faster for finger responses compared to leg responses (MD = 447ms). The stimulus location × response side interaction was significant, F(1, 31) = 27.95, *p* < .001, ηp² = 0.47 (ηG² = 0.065). This interaction reflects the Simon effect, with participants responding significantly faster when stimulus location and response side were congruent — a pattern further decomposed into four response-driven and stimulus-driven contrast types in the analyses below. No other two-way interactions reached significance.

**Fig. 3:**
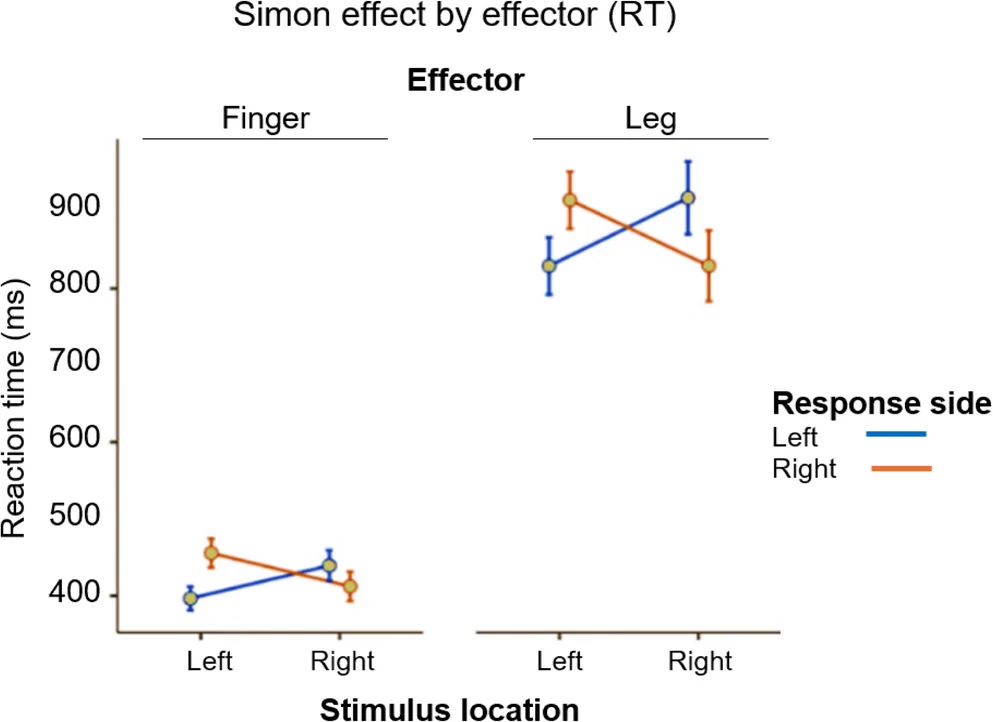
Simon effect on reaction time (ms). Estimated marginal means for reaction time (ms) are shown as a function of stimulus location, response side, and effector, illustrating the interaction pattern underlying the Simon effect across finger and leg responses

A significant three-way interaction among stimulus location, response side, and effector, F(1, 31) = 18.13, *p* < .001, ηp² = 0.37 (ηG² = 0.013), suggests that the magnitude of the Simon effect differed across effectors. Post hoc Bonferroni comparisons revealed significant Simon effects for all contrast types in finger responses except the right response-driven contrast (right–left versus right–right; MD = 27.0 ms, *p* = .078; see Table 1; Fig. 3). For leg responses, all contrasts were significant, with consistently larger interference than for finger responses.

To contextualize the four-type decomposition within the conventional framework, a classic Simon effect analysis was also conducted. Congruent RT was defined as the mean of left stimulus / left response and right stimulus / right response trials; incongruent RT as the mean of left stimulus / right response and right stimulus / left response trials, averaged across previous-trial congruency. Finger responses produced a Simon effect of + 43.02 ms (*SD* = 27.03, 95% CI [33.66, 52.38]), *t*(31) = 9.004, *p* < .001, *d* = 1.592, and leg responses produced a Simon effect of + 87.01 ms (*SD* = 54.39, 95% CI [68.17, 105.86]), *t*(31) = 9.049, *p* < .001, *d* = 1.600. Although the leg Simon effect was significantly larger in absolute terms (+ 43.99 ms, t(31) = 4.258, *p* < .001, d = 0.753), both effectors produced virtually identical proportional interference relative to their congruent RT baseline—calculated as (RT_incongruent − RT_congruent) / RT_congruent × 100—(10.64% for fingers, 10.49% for legs), indicating that spatial compatibility effects are similarly scaled across the two motor systems when baseline speed differences are taken into account.

**Table 1:** Simon effect (ms) by effector. Post hoc comparisons for the interaction between stimulus position, response side, and effector. The causes of conflict are indicated in bold

Effector	Stimulus location	Response side	vs.	Stimulus location	Response side	Difference Mean	SE	t	Bonferroni
Finger	left	**Right**	-	left	Left	58.9	6.23	9.45	**< 0.001**
	right	**Left**	-	right	Right	27.0	8.34	3.24	0.078
	**right**	Left	-	left	Left	42.7	6.32	6.76	**< 0.001**
	**left**	Right	-	right	Right	43.2	6.12	7.06	**< 0.001**
	left	Right	-	right	Left	16.1	6.39	2.53	0.465
	right	Right	-	left	Left	15.6	7.33	2.13	1.000
Leg	left	**Right**	-	left	Left	85.6	15.37	5.57	**< 0.001**
	right	**Left**	-	right	Right	88.3	15.33	5.76	**< 0.001**
	**right**	Left	-	left	Left	88.5	15.10	5.86	**< 0.001**
	**left**	Right	-	right	Right	85.5	11.73	7.28	**< 0.001**
	right	Left	-	left	Right	2.8	14.01	0.20	1.000
	right	Right	-	left	Left	0.1	16.46	0.009	1.000

df = 31

The analysis also revealed a significant three-way interaction between stimulus position, response side, and previous-trial congruency, *F*(1, 31) = 44.45, *p* < .001, ηp² = 0.589 (ηG² = 0.018). This indicates a CSE; the Simon effect was reduced following incongruent relative to congruent previous trials. Bonferroni comparisons confirmed that the Simon effect was strongly influenced by previous-trial congruency for both effectors (see Tables 2 and 3). As expected, after congruent trials, the Simon effect was large and reliable (finger: ≈54–86 ms; leg: ≈108–115 ms, all ps < 0.001) but was significantly reduced following incongruent trials. However, the four-way interaction between stimulus position, response side, previous congruence, and effector was not significant, *F*(1, 31) = 0.09, *p* = .770, ηp² = 0.003 (ηG² ≈ 0.000), indicating that the influence of previous-trial congruency on the Simon effect did not differ significantly between effectors.

**Fig. 4:**
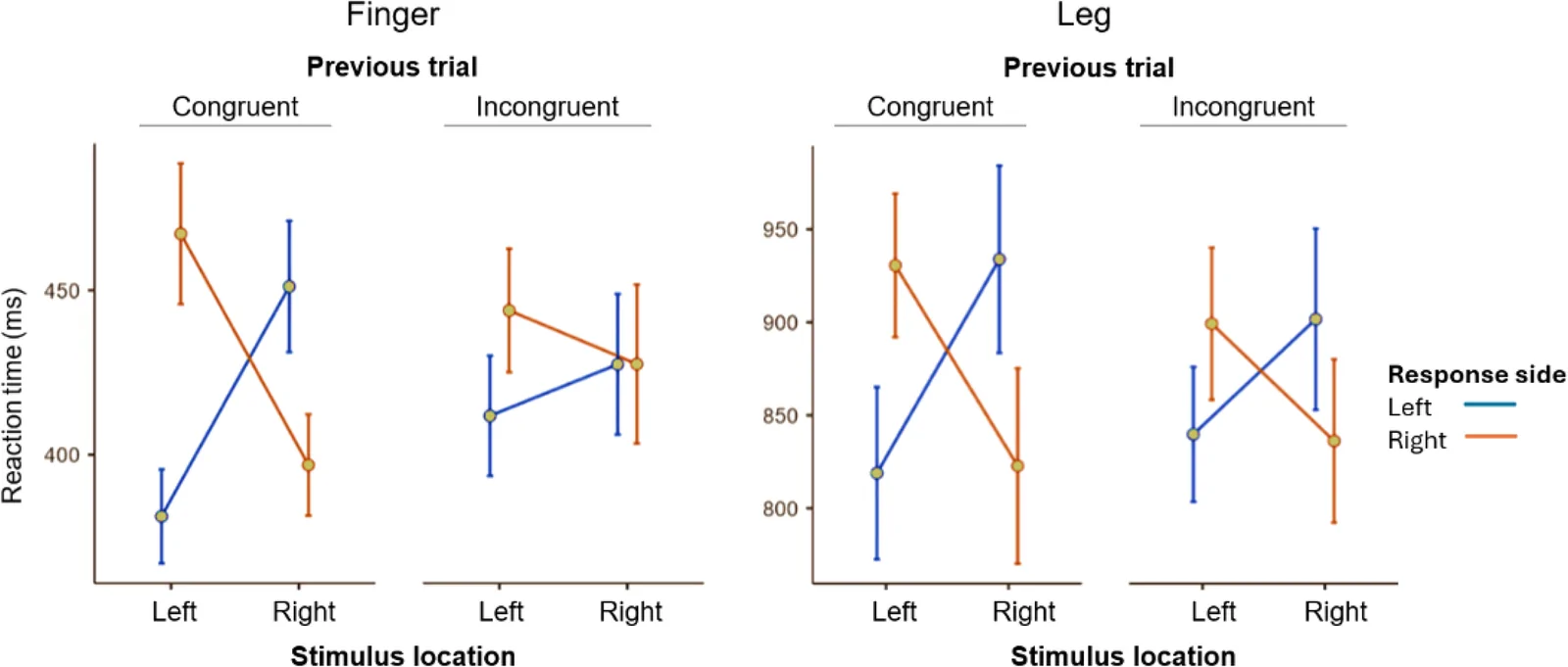
Sequential effects on reaction time (ms). Estimated marginal means by stimulus location, response side, and previous-trial condition, illustrating the sequential effect. Error bars represent 95% confidence intervals

To examine this pattern of effects within each effector independently, two additional three-way repeated-measures ANOVAs were subsequently conducted — one for finger responses and one for leg responses — each with stimulus position (left, right), response side (left, right), and previous-trial congruency (congruent, incongruent) as within-subjects factors. Specifically, for finger responses, a significant Simon effect was observed only in the right response-driven interference (left–right vs. left–left; ≈32 ms, *p* = .026), whereas all other contrasts were nonsignificant. For leg responses, the effect remained significant for left response-driven interference (right–left versus right–right ≈ 66 ms, *p* = .022) and left stimulus-driven interference (left–right versus right–right ≈ 63 ms, *p* = .004) but was nonsignificant for the other contrasts (ps ≥ 0.148). This CSE corresponding to a 77.2% reduction in the Simon effect for finger responses (M = + 54.11 ms, SD = 39.91, 95% CI [40.28, 67.94], t(31) = 7.670, *p* < .001, d = 1.356) and a 43.9% reduction for leg responses (M = + 48.96 ms, SD = 84.26, 95% CI [19.77, 78.15], t(31) = 3.287, *p* < .01, d = 0.581), calculated as 1 − (Simon effect after incongruent previous trial / Simon effect after congruent previous trial), averaged across the four effect types (see Figs. 4).

**Table 2:** Sequential effects for reaction time (ms) in finger responses. Post hoc comparisons for the interaction between stimulus position, response side, and previous trial

Previous trial	Stimulus location	Finger response	-	Stimulus location	Finger response	Mean Difference	SE	t	Bonferroni
Congruent	left	**Right**	-	left	Left	85.88	7.12	12.065	**< 0.001**
	right	**Left**	-	right	Right	54.26	7.96	6.815	**< 0.001**
	**right**	Left	-	left	Left	69.86	7.26	9.619	**< 0.001**
	**left**	Right	-	right	Right	70.28	5.89	11.939	**< 0.001**
	left	Right	-	right	Left	16.01	6.09	2.630	0.369
	right	Right	-	left	Left	15.60	7.11	2.194	1.000
Incongruent	left	**Right**	-	left	Left	32.02	8.74	3.663	**0.026**
	right	**Left**	-	right	Right	-0.09	10.62	-0.008	1.000
	**right**	Left	-	left	Left	15.65	8.06	1.942	1.000
	**left**	Right	-	right	Right	16.26	9.53	1.707	1.000
	left	Right	-	right	Left	16.36	9.23	1.772	1.000
	right	Right	-	left	Left	15.75	9.56	1.647	1.000

**Table 3:** Sequential effects (ms) in leg responses. Post hoc comparisons for the interaction between stimulus position, response side, and previous trial

Previous Trial	Stimulus location	Leg response	vs.	Stimulus location	Leg response	Mean Difference	SE	t	Bonferroni
Congruent	left	**Right**	-	left	Left	111.79	17.5	6.370	**< 0.001**
	right	**Left**	-	left	Left	115.08	16.0	7.184	**< 0.001**
	**left**	Right	-	right	Right	107.90	15.3	7.034	**< 0.001**
	**right**	Left	-	right	Right	111.20	19.0	5.849	**< 0.001**
	right	Right	-	left	Left	3.88	20.8	0.187	1.000
	right	Left	-	left	Right	3.29	16.8	0.196	1.000
Incongruent	left	**Right**	-	left	Left	59.53	20.5	2.899	0.191
	right	**Left**	-	right	Right	65.54	17.6	3.716	**0.022**
	**left**	Right	-	right	Right	63.11	14.5	4.345	**0.004**
	**right**	Left	-	left	Left	61.96	20.7	2.999	0.148
	left	Left	-	right	Right	3.58	18.2	0.196	1.000
	right	Left	-	left	Right	2.43	17.4	0.140	1.000

### Errors Simon effect

Error trials were included, and the same four-way repeated measures ANOVA was conducted on percentage errors (PE). The analysis revealed a large main effect of effector, F(1, 31) = 129.87, *p* < .001, ηp² = 0.81 (ηG² = 0.166), with higher error rates for finger than leg responses (MD = 4.49%). There was also a significant main effect of previous trial, F(1, 31) = 6.34, *p* = .017, ηp² = 0.17 (ηG² = 0.014), with more errors following incongruent than congruent trials (MD = 1.19%, *p* = .017). The stimulus location × response side interaction was significant, F(1, 31) = 27.95, *p* < .001, ηp² = 0.47 (ηG² = 0.065), consistent with a Simon effect. Errors were more frequent when stimulus location and response side were incongruent than when they were congruent.

**Fig. 5:**
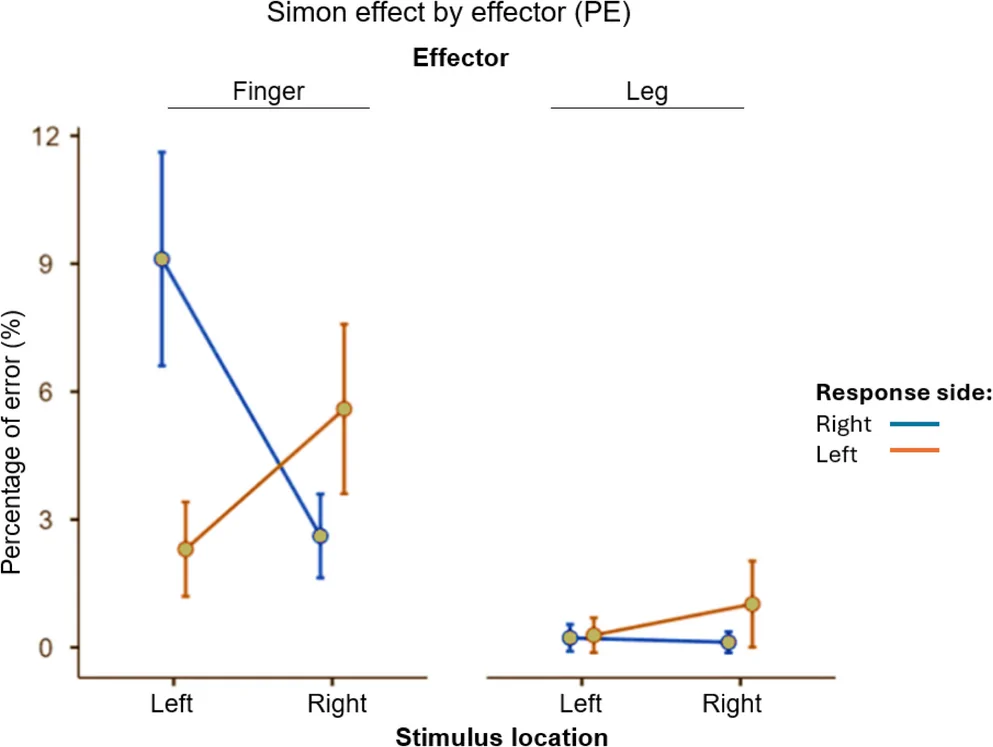
Simon effects on the percentage of errors. Estimated marginal means for percentage of errors as a function of stimulus location, response side, and effector, illustrating the interaction pattern underlying the Simon effect across finger and leg responses. Error bars represent 95% confidence intervals

A significant three-way interaction of stimulus location × response side × effector, F(1, 31) = 25.13, *p* < .001, ηp² = 0.45 (ηG² = 0.047), indicated that the magnitude of the Simon effect differed across effectors (See Fig. 5). Post-hoc comparisons revealed that, for finger responses, participants made more errors on right response-driven (MD = 6.81%, SE = 1.37, t = 4.96, *p* < .001) and left stimulus-driven (MD = 6.50%, SE = 1.26, t = 5.17, *p* < .001) contrasts. Other contrasts for finger responses were nonsignificant after Bonferroni correction (ps ≥ 0.299). In contrast, for leg responses, none of the Simon contrasts reached significance (all ps = 1.00), indicating that error-based Simon effects were evident for finger responses but not for leg responses (see Table 4).

**Fig. 6:**
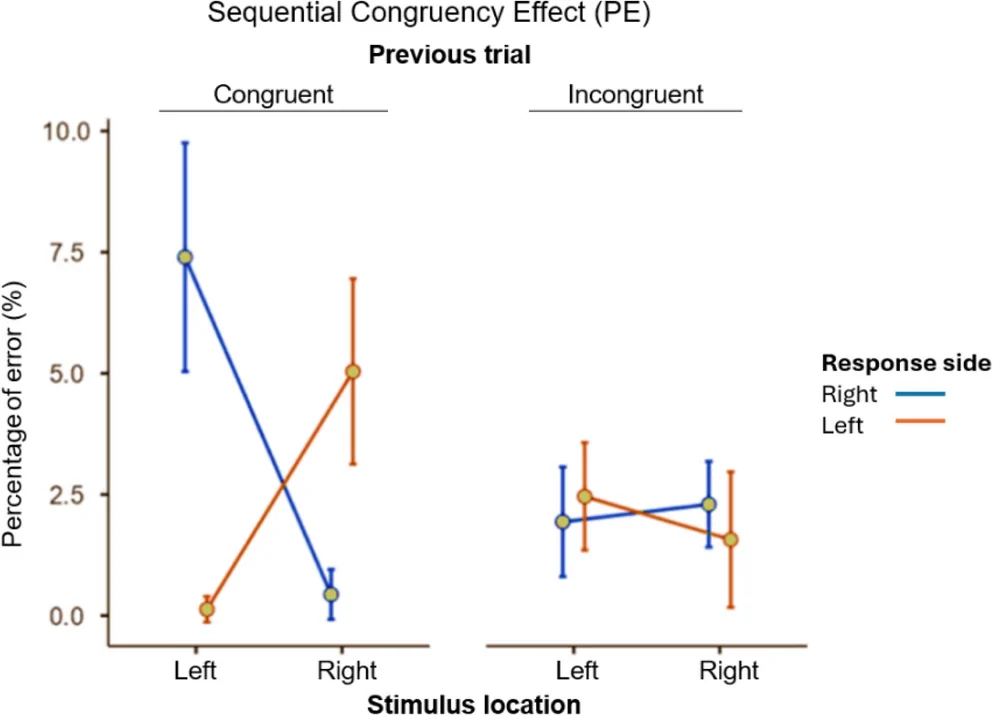
Sequential effects on the percentage of errors. Estimated marginal means as a function of stimulus location, response side, and previous-trial condition, illustrating conflict adaptation effects

There was also a significant Stimulus location × response side × previous trial interaction, F(1, 31) = 63.45, *p* < .001, ηp² = 0.67 (ηG² = 0.096), indicating a CSE in error rates. (see Fig. 6). The four-way interaction **(**Stimulus × Response × Effector × Previous trial**)** was also significant, *F* (1, 31) = 59.10, *p* < .001, ηp² = 0.062 (ηG² = 0.090), indicating that the effect of trial history on the Simon effect differed across effectors (see Tables 5 and 6). Follow-up analyses conducted separately for each effector type revealed that, for finger responses, significant Simon effects in error rates were observed following congruent trials, whereas no Simon contrasts reached significance following incongruent trials (all ps = 1.00). In contrast, leg responses showed no significant Simon effects following either congruent or incongruent trials (all ps = 1.00), indicating that congruency-sequence modulation of error-based Simon effects was restricted to finger responses (see Tables 5 and 6).

**Table 4:** Error-based Simon effects by effector. Post hoc comparisons for the interaction between stimulus position, response side, and effector

Effector	Stimulus location	Response side	Vs	Stimulus location	Response side	Mean Difference	SE	t	Bonferroni
Finger	left	**Right**	-	left	Left	6.80	1.371	4.963	**< 0.001**
	right	**Left**	-	right	Right	2.97	1.096	2.718	0.299
	**right**	Left	-	left	Left	3.28	1.299	2.532	0.465
	**left**	Right	-	right	Right	6.49	1.257	5.167	**< 0.001**
	left	Right	-	right	Left	3.51	1.530	2.299	0.795
	left	Left	-	right	Right	-0.31	0.756	-0.412	1.000
leg	left	**Left**	-	left	Right	0.06	0.261	0.242	1.000
	left	**Right**	-	right	Right	0.10	0.112	0.918	1.000
	**left**	Left	-	right	Left	-0.73	0.408	-1.787	1.000
	**right**	Right	-	right	Left	-0.89	0.517	-1.732	1.000
	left	Right	-	right	Left	-0.79	0.533	-1.488	1.000
	left	Left	-	right	Right	0.16	0.238	0.698	1.000

**Table 5:** Error-based sequential effects in finger responses. Post hoc comparisons for the interaction between stimulus position, response side, and previous trial

Previous trial	Stimulus location	Response side	Vs	Stimulus location	Response side	Mean Difference	SE	t	Bonferroni
Congruent	left	**Right**	-	left	Left	14.34	2.21	6.47	**< 0.001**
	right	**Left**	-	right	Right	8.33	1.79	4.64	**0.007**
	**left**	Right	-	right	Right	13.47	2.26	5.94	**< 0.001**
	**right**	Left	-	left	Left	9.21	1.67	5.51	**< 0.001**
	left	Right	-	right	Left	5.13	2.71	1.89	1.000
	right	Right	-	left	Left	0.87	0.50	1.73	1.000
Incongruent	left	**Right**	-	left	Left	-0.73	1.46	-0.50	1.000
	right	**Left**	-	right	Right	-2.38	1.12	-2.11	1.000
	**left**	Right	-	right	Right	-0.48	1.45	-0.33	1.000
	**right**	Left	-	left	Left	-2.63	1.37	-1.92	1.000
	left	Left	-	right	Right	0.25	1.45	0.17	1.000
	left	Right	-	right	Left	1.89	1.39	1.36	1.000

**Table 6:** Error-based sequential effects in leg responses. Post hoc comparisons for the interaction between stimulus position, response side, and previous trial. Across both previous-trial conditions, no Simon contrasts reached significance

Previous trial	Stimulus location	Response side	Vs	Stimulus location	Response side	Mean Difference	SE	t	Bonferroni
Congruent	left	**Right**	-	left	Left	0.186	0.414	0.44	1.000
	right	**Left**	-	right	Right	0.868	0.490	1.77	1.000
	**right**	Left	-	left	Left	-0.607	0.568	-1.06	1.000
	**left**	Right	-	right	Right	0.446	0.311	1.43	1.000
	left	Right	-	right	Left	-0.421	0.601	-0.70	1.000
	left	Left	-	right	Right	0.260	0.260	1.00	1.000
Incongruent	left	**Right**	-	left	Left	-0.312	0.312	-1.00	1.000
	right	**Left**	-	right	Right	0.924	0.940	0.983	1.000
	**left**	Right	-	right	Right	-0.240	0.240	-1.00	1.000
	**right**	Left	-	left	Left	0.852	0.615	1.38	1.000
	left	Right	-	right	Left	-1.164	0.898	-1.29	1.000
	left	Left	-	right	Right	0.072	0.400	0.180	1.000

### Simon effect across blocks

We also investigated practice-related changes in spatial conflict resolution by including block (1, 3) as a factor in the 2 (stimulus position) × 2 (response side) × 2 (effector) × 2 (block) repeated-measures ANOVA on RTs. The main effect of block was not significant, *F*(1, 31) = 0.03, *p* = .870, η*p*² = 0.001 (η*G*² = 0.000). There was a significant stimulus position × response side × block interaction, *F*(1, 31) = 8.09, *p* = .008, η*p*² = 0.207 (η*G*² = 0.008), indicating that the Simon effect changed across blocks. This was further qualified by a significant four-way interaction among stimulus position, response side, block, and effector, *F*(1, 31) = 5.15, *p* = .030, η*p*² = 0.142 (η*G*² = 0.006), indicating that practice-related changes in conflict resolution differed between effectors (see Tables 7 and 8). To decompose this interaction, post hoc analyses were conducted separately for each effector. To characterize block-wise changes, absolute and proportional Simon effects were also calculated across all three blocks using the block-level data, with Block 2 reported descriptively because it was excluded from the primary ANOVA to reduce model complexity and minimize sphericity violations.

For finger responses, three of the four contrasts were significant in both Block 1 — right response-driven (65.6 ms), right stimulus-driven (41.1 ms), and left stimulus-driven (53.7 ms; all *p*s < 0.001) — and Block 3 (51.0, 49.0, and 37.6 ms respectively; *p*s ≤ 0.009), with the left response-driven contrast non-significant in both blocks (*p*s ≥ 0.193). The overall pattern was one of modest and uneven change: the right response-driven and left stimulus-driven contrasts decreased modestly (~ 22% and ~ 30% respectively), whereas the right stimulus-driven contrast increased slightly (41.1 → 49.0 ms). The classic Simon effect (computed as the mean incongruent–congruent RT difference across the four contrast pairs) was 47.5 ms in Block 1, 44.6 ms in Block 2, and 43.4 ms in Block 3 — an absolute reduction of only 4.1 ms (~ 9%) across the session. Expressed relative to the congruent RT baseline, proportional interference showed an equivalently shallow trajectory: 11.77% (Block 1), 11.03% (Block 2), and 10.76% (Block 3), indicating that finger-based spatial conflict resolution was largely stable across practice.

**Fig. 7:**
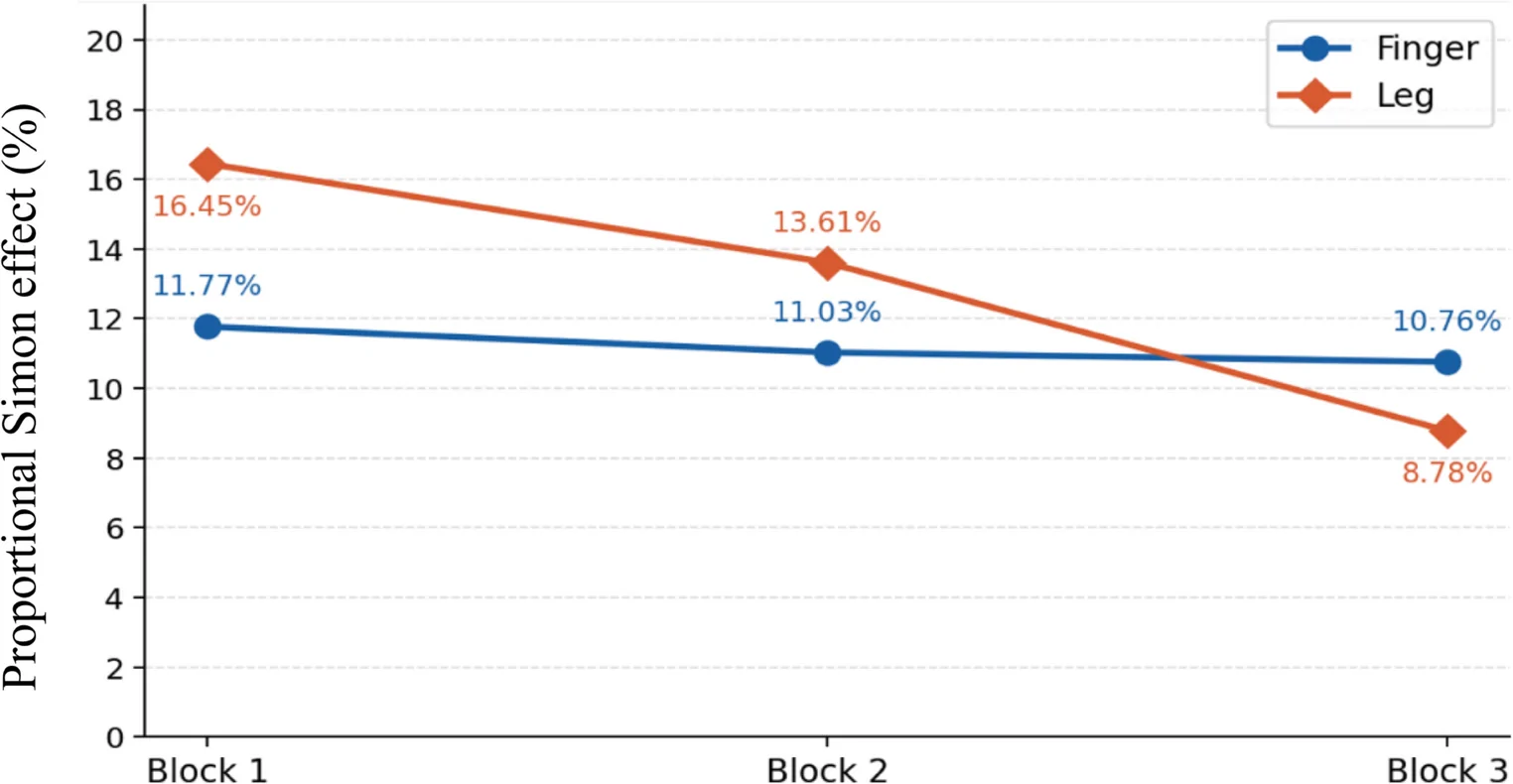
Proportional Simon effect across practice blocks for finger and leg responses. Proportional interference was computed as $\:({RT}_{incongruent}-{RT}_{congruent})/{RT}_{congruent}\times\:100$, using the congruent reaction time for each effector as the baseline. Finger responses exhibited relatively stable proportional interference across blocks (11.77%, 11.03%, and 10.76%), whereas leg responses showed a pronounced monotonic decline (16.45%, 13.61%, and 8.78%), falling below the finger level by Block 3. Although Block 2 was excluded from the primary ANOVA to reduce model complexity and mitigate sphericity concerns, it is included here descriptively to illustrate the trajectory of practice-related change

For leg responses, all four contrasts were large and significant in Block 1 (119.7–142.4 ms, all *p*s < 0.001) but decreased substantially in Block 3 (71.1–74.6 ms), with only the right stimulus-driven contrast remaining significant (*MD* = 74.6 ms, *p* = .016). The classic Simon effect was 131.1 ms in Block 1, 110.6 ms in Block 2, and 72.9 ms in Block 3 — an absolute reduction of 58.2 ms (~ 44%) across the session, with the largest drop occurring between Blocks 2 and 3. Proportional interference followed a corresponding monotonic decline: 16.45% (Block 1), 13.61% (Block 2), and 8.78% (Block 3) — a total reduction of 7.67% points, falling below the finger proportional level by Block 3 (see Fig. 7). The progressive nature of this decline across all three blocks is consistent with practice-related adaptation rather than a one-off adjustment, and indicates that the block effect reflects attenuation of spatial compatibility scaling rather than simply reflecting the larger baseline interference present at the start of the session. The same four-way ANOVA for error rates showed that the main effect of block was not significant, *F*(1, 31) = 3.62, *p* = .066, η*p*² = 0.105 (η*G*² = 0.007), and all block-related interactions were also non-significant (all *p*s ≥ 0.088).

**Table 7:** Simon effects (ms) for finger responses across Block 1 and Block 3. Simon effects, as a function of stimulus location and response side, showed minimal change from Block 1 to Block 3 but varied across effect types

Block	Stimulus location	Response side	Vs	Stimulus location	Response side	Mean Difference	SE	t	Bonferroni
1	left	**Right**	-	left	Left	65.6	8.52	7.69	**< 0.001**
	right	**Left**	-	right	Right	29.2	8.47	3.45	0.193
	**right**	Left	-	left	Left	41.1	5.43	7.58	**< 0.001**
	**left**	Right	-	right	Right	53.7	9.02	5.95	**< 0.001**
	left	Left	-	right	Right	-11.8	7.58	-1.56	1.000
	left	Right	-	right	Left	24.4	6.83	3.58	0.139
3	left	**Right**	-	left	Left	51.0	8.95	5.70	**< 0.001**
	right	**Left**	-	right	Right	35.6	11.40	3.12	0.458
	**right**	Left	-	left	Left	49.0	9.99	4.91	**0.003**
	**left**	Right	-	right	Right	37.6	8.28	4.54	**0.009**
	left	Left	-	right	Right	-13.4	6.85	-1.96	1.000
	left	Right	-	right	Left	1.981	10.88	0.18	1.000

**Table 8:** Simon effects (ms) for leg responses across Block 1 and Block 3. Simon effects were significantly reduced in Block 3 compared with Block 1, with only the right stimulus-driven effect remaining significant

Block	Stimulus location	Response side	Vs	Stimulus location	Response side	Mean Difference	SE	t	Bonferroni
1	left	**Right**	-	left	Left	142.4	21.0	6.761	**< 0.001**
	right	**Left**	-	right	Right	119.7	16.1	7.398	**< 0.001**
	**right**	Left	-	left	Left	141.4	14.9	9.446	**< 0.001**
	**left**	Right	-	right	Right	120.7	14.3	8.395	**< 0.001**
	left	Left	-	right	Right	-21.7	16.9	-1.27	1.000
	left	Right	-	right	Left	0.97	16.0	0.061	1.000
3	left	**Right**	-	left	Left	71.8	21.2	3.37	0.239
	right	**Left**	-	right	Right	74.0	24.3	3.039	0.575
	**right**	Left	-	left	Left	74.6	17.1	4.365	**0.016**
	**left**	Right	-	right	Right	71.1	20.4	3.483	0.180
	left	Left	-	right	Right	-0.6	22.9	-0.026	1.000
	left	Right	-	right	Left	-2.8	20.4	-0.138	1.000

### Classic Simon effect

To contextualize the four-type decomposition within the conventional framework, a classic Simon effect analysis was also conducted. Congruent RT was defined as the mean of left stimulus / left response and right stimulus / right response trials; incongruent RT as the mean of left stimulus / right response and right stimulus / left response trials, averaged across previous-trial congruency. Finger responses produced a Simon effect of + 43.02 ms (*SD* = 27.03, 95% CI [33.66, 52.38]), *t*(31) = 9.004, *p* < .001, *d* = 1.592, and leg responses produced a Simon effect of + 87.01 ms (*SD* = 54.39, 95% CI [68.17, 105.86]), *t*(31) = 9.049, *p* < .001, *d* = 1.600. Although the leg Simon effect was significantly larger in absolute terms (+ 43.99 ms, t(31) = 4.258, *p* < .001, d = 0.753), both effectors produced virtually identical proportional interference relative to their congruent RT baseline—calculated as (RT_incongruent − RT_congruent) / RT_congruent × 100—(10.64% for fingers, 10.49% for legs), indicating that spatial compatibility effects are similarly scaled across the two motor systems when baseline speed differences are taken into account.

### Two-way congruency sequence effect

To address the standard conflict adaptation framework (Gratton et al., 1992), the CSE was examined as the interaction between current-trial compatibility (compatible vs. incompatible) and previous-trial congruency (congruent vs. incongruent), computed separately for each effector. For both finger and leg responses, the Simon effect was substantially larger following a congruent previous trial than following an incongruent previous trial, confirming a robust CSE. For finger responses, the Simon effect decreased from 70.07 ms (SD = 30.17) following congruent previous trials to 15.96 ms (SD = 36.70) following incongruent previous trials, representing an absolute reduction of 54.11 ms (SD = 39.91, 95% CI [40.28, 67.94]), t(31) = 7.670, *p* < .001, d = 1.356, F(1, 31) = 58.83, η²*p* = .655. Although markedly attenuated, the residual Simon effect after incongruent previous trials remained significant, t(31) = 2.461, *p* = .020, d = 0.435, indicating that conflict adaptation reduced spatial compatibility interference by 77.2% but did not eliminate it completely. For leg responses, the Simon effect decreased from 111.49 ms (SD = 59.90) following congruent previous trials to 62.53 ms (SD = 76.68) following incongruent previous trials, corresponding to an absolute reduction of 48.96 ms (SD = 84.26, 95% CI [18.58, 79.34]), t(31) = 3.287, *p* = .003, d = 0.581, F(1, 31) = 10.81, η²*p* = .258. The residual Simon effect also remained significant, t(31) = 4.613, *p* < .001, d = 0.816. However, because the initial Simon effect was considerably larger for legs, this reduction represented a smaller proportional attenuation (43.9%) than that observed for fingers.

Critically, the magnitude of the CSE, defined as the absolute reduction in the Simon effect following incongruent relative to congruent previous trials, did not differ significantly between effectors, F(1, 31) = 0.09, *p* = .770, η²*p* = .003, 95% CI [− 40.72, 30.42]. Thus, finger and leg responses exhibited equivalent trial-by-trial conflict adaptation when assessed in absolute milliseconds, despite the larger baseline Simon effect and correspondingly greater residual interference observed for leg responses. This pattern complements the four-type decomposition reported above by indicating that sequential conflict adaptation operates similarly across effectors, even though the underlying magnitude and composition of spatial interference differ between finger and whole-body responses.

## Discussion

The present findings challenge the assumption of a unitary Simon effect, demonstrating that spatial conflict resolution is shaped—at least in part—by distinct, effector-dependent structures and adaptive dynamics across manual and lower-limb response systems. Rather than indexing a single, homogeneous form of interference, the conventional aggregate Simon effect masks systematically different patterns that emerge when stimulus- and response-related contributions are disentangled. In particular, standing leg responses showed significant interference across all four decomposed contrasts and a pronounced reduction in interference with practice, whereas finger responses exhibited a more lateralized and temporally stable interference profile, alongside stronger trial-by-trial sequential adaptation. Together, these results provide evidence that the Simon effect is better conceptualized as a composite of separable stimulus- and response-driven components, and that the motor effector engaged shapes the organisation, adaptive modulation, and practice-related plasticity of spatial conflict processing.

### Simon effect: reaction time

This study applied an established analytical approach that treats stimulus location and response side as independent factors (Chen et al., 2021; Proctor et al., 2023; Tagliabue et al., 2007). This framework captures the classic Simon effect via the stimulus × response interaction while also enabling direct testing of lateralized asymmetries across both stimulus and response dimensions. The present study extended this model by decomposing the Simon effect into four directional contrasts, partitioning the stimulus–response compatibility space into distinct sources of interference rather than treating it as a single congruency effect. This decomposition provides a framework for quantifying effector-dependent asymmetries in spatial interference that are not captured by conventional collapsed measures.

Consistent with previous findings (Gupta et al., 2022; Leuthold & Schröter, 2006; Mittelstädt & Miller, 2020; Proctor et al., 2023), leg responses exhibited larger absolute Simon effects than finger responses across all four contrast types. However, when expressed as a proportion of each effector’s congruent RT baseline, both effectors produced virtually identical relative interference (10.64% for fingers and 10.49% for legs), suggesting that the absolute difference reflects the slower RT baseline of the leg effector rather than disproportionately stronger spatial coding. This proportional equivalence is consistent with a motor-constraint account in which a slower effector naturally accumulates more automatic spatial activation before conflict resolution occurs (Ulrich et al., 2015) and cautions against interpreting the absolute RT difference as unambiguous evidence of qualitatively distinct cognitive-control processes.

Crucially, while a global index implies that spatial compatibility is merely scaled identically across motor systems, the subsequent elemental decomposition and block-wise analysis unmask radically divergent internal configurations between the limbs. Separate analyses revealed a clear dissociation between effectors in the internal structure of spatial interference. For finger responses, the four contrasts were asymmetrically distributed, with the right response-driven effect being the largest (58.9 ms) and the left response-driven effect the smallest and non-significant (27.0 ms), whereas leg responses exhibited significant and broadly comparable interference across all four contrasts, suggesting a more bilaterally organized spatial coding architecture. Notably, the asymmetry within finger responses was confined to the response-driven contrasts (58.9 ms vs. 27.0 ms), with no meaningful asymmetry between the stimulus-driven contrasts (42.7 ms vs. 43.2 ms), providing behavioural evidence that the effector-specific lateralisation mainly originates at the level of response selection rather than perceptual encoding — a pattern consistent with a two-locus architecture of the Simon effect (Cespón et al., 2020; Stürmer et al., 2002). Computational modelling indicates that hemispheric asymmetries in attentional orienting, rather than response-selection processes alone, are the primary driver of lateralised Simon effect patterns — though the specific direction of asymmetry may depend on visual angle and task configuration (Spironelli et al., 2009).

**Fig. 8:**
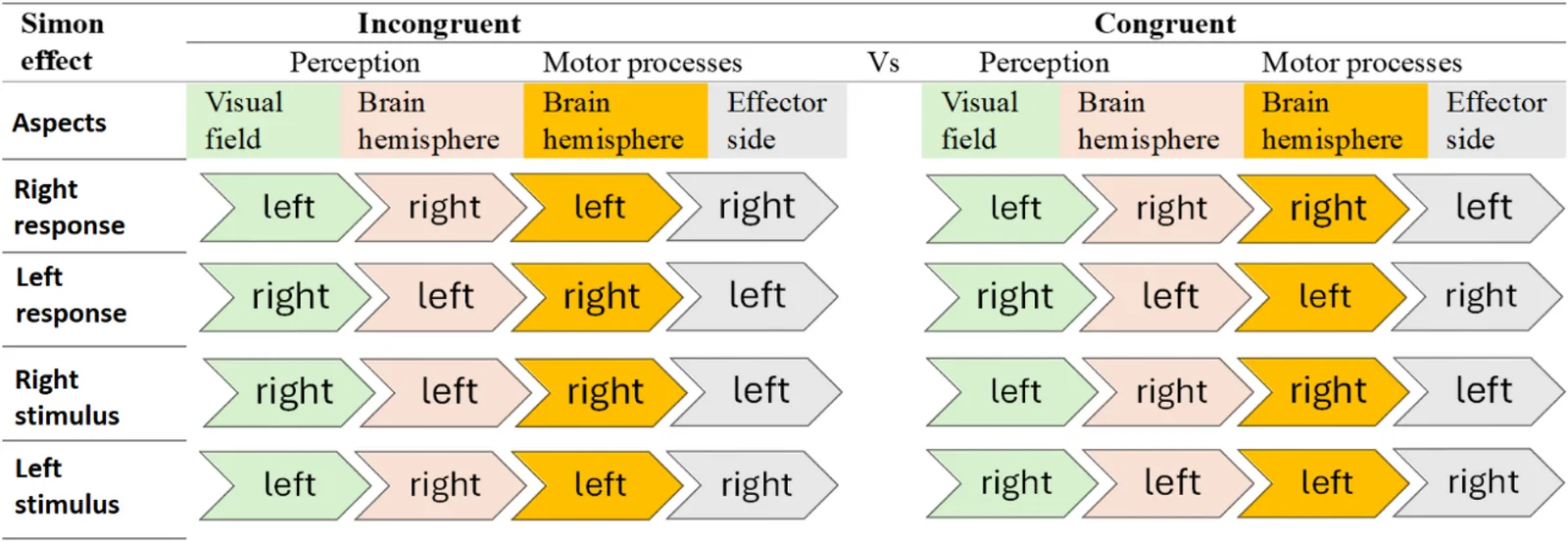
Conceptual framework of spatial interference in the Simon task. Schematic illustration of four distinct forms of spatial interference arising from the independent contributions of stimulus location (perceptual–attentional; stimulus-driven) and response side (motor; response-driven) under congruent and incongruent conditions. In incongruent trials, misalignment between stimulus location and required response side generates spatial conflict either at the perceptual level, through competition between visual fields and hemispheric processing, or at the motor level, through cross-hemispheric activation of competing response representations. In contrast, congruent trials involve alignment of perceptual and motor spatial codes, reducing the need for cross-hemispheric integration and minimizing conflict. The schematic emphasizes that the Simon effect arises from the interaction of separable perceptual and motor spatial representations and illustrates how their relative contributions may vary across effectors and task contexts

The lateralized asymmetry observed for finger responses can be interpreted within a perceptual–motor lateralization framework in which spatial interference reflects a mismatch between stimulus-driven hemispheric activation and response execution demands (see Fig. 8). Left and right stimulus locations engage partially distinct neurocognitive mechanisms: stimuli in the left visual field elicit stronger and faster attentional orienting than those in the right visual field—a phenomenon known as pseudoneglect—driven by the right-hemisphere ventral attention network responsible for bottom-up attentional capture and spatial reorienting (Cook et al., 2025; Corbetta et al., 2008; DiNuzzo et al., 2022; Shulman et al., 2010). This enhanced processing generates a stronger automatic spatial code for left-field stimuli, facilitating congruent left responses but increasing interference when this activation must be overridden to produce an incongruent right-sided response.

Nevertheless, this attentional asymmetry does not necessarily translate into greater spatial interference for left-sided stimuli (Proctor et al., 2023; Rubichi & Nicoleti, 2006; Spironelli et al., 2009). Right-handed individuals are often found to exhibit a more pronounced Simon effect for right-sided stimuli (typically at ~ 4.5° visual angle) when responding with either their fingers or legs (Chen et al., 2021; Seibold et al., 2016). This has been explained by findings that non-corresponding responses to right-sided stimuli are executed with the slower left effector, while corresponding responses engage the faster right effector, leading to a larger Simon effect (Chen et al., 2021; Proctor et al., 2023). The present study used a wider visual angle of 11.5°, which may have amplified hemispheric asymmetries in attentional capture and shifted the dominant source of interference toward left visual field stimuli, consistent with the pseudoneglect account outlined above. Whether visual angle is the critical moderating factor warrants direct empirical investigation, as the direction of Simon effect asymmetry may also depend on effector type and the visibility of response devices during task performance (Chen et al., 2021; Proctor et al., 2023; Wallace, 1972).

### Simon effect: error rates

A parallel pattern emerged in error rates, providing convergent support for the RT findings. The significant three-way interaction among stimulus location, response side, and effector indicated that error-based Simon effects differed across effectors. For finger responses, two contrasts reached significance after Bonferroni correction: the right response-driven effect (6.81%) and the left stimulus-driven effect (6.50%), while the left response-driven (2.97%) and right stimulus-driven (3.28%) contrasts did not survive correction. Critically, these two significant error contrasts — right response-driven and left stimulus-driven — are precisely the same contrasts that produced the largest effects in RT (58.9 ms and 43.2 ms respectively), and both involve left visual field input. This convergence across two independent measures strengthens the hemispheric lateralization account: the contrasts driven by left visual field stimuli are robustly significant in both speed and accuracy, whereas right visual field contrasts are either marginal or absent across both measures. For leg responses, none of the four contrasts reached significance in errors, consistent with the near-zero error rates observed for this effector (M = 0.41% vs. 4.90% for fingers).

The dissociation between effectors in error rates — larger absolute RT interference for legs but significantly higher error rates for fingers — suggests that the two effector systems operate under qualitatively different speed–accuracy regimes. The longer movement times associated with leg responses appear to afford an extended window for goal-directed cognitive control to suppress spatially driven response tendencies before the action is completed, permitting error correction that is unavailable to the faster finger system (Volz et al., 2015). This pattern is consistent with a cautious, accuracy-prioritizing control strategy in the lower-limb motor system, in contrast to the faster but more error-prone upper-limb system where spatial pull errors are more likely to escape suppression before response completion.

### Congruency sequence effect: reaction time

Both effectors exhibited a significant CSE, with Simon interference reduced following incongruent relative to congruent previous trials. This extends previous demonstrations of sequential conflict adaptation in manual Simon tasks (Botvinick et al., 2001; Cespón et al., 2020; Gratton et al., 1992; Stürmer et al., 2002; Wühr & Ansorge, 2005) by showing that trial-by-trial modulation of spatial interference also occurs during lower-limb responding, indicating that sequential conflict adaptation generalizes beyond upper-limb effectors.

Importantly, the four-way interaction involving effector and previous-trial congruency was not significant, indicating no reliable difference in the absolute magnitude of conflict adaptation between finger and leg responses. However, this null effect should be interpreted with caution given the limited statistical power of the sequential analyses. Follow-up analyses conducted separately for each effector showed that, although overall adaptation was statistically comparable, its internal expression across stimulus–response mappings differed systematically: finger responses exhibited near-complete attenuation of interference, whereas leg responses retained residual interference in specific contrast types. This pattern is not captured by aggregate interaction terms and suggests that sequential conflict resolution may be effector-dependent in its functional organization rather than fully uniform across motor systems, consistent with configuration-specific modulation of spatial interference rather than a unitary, effector-neutral control signal. Critically, although the absolute magnitude of adaptation was similar across effectors, its proportional effectiveness differed, as the same reduction in interference translated into a smaller proportional attenuation for legs due to their larger baseline interference (43.9% vs. 77.2% for fingers).

The mechanisms underlying the CSE remain debated: conflict-monitoring accounts attribute sequential reductions to dynamic upregulation of cognitive control following high-conflict trials (Botvinick et al., 2001; Ridderinkhof, 2002), whereas feature integration and event-file frameworks emphasize stimulus–response binding and repetition-based mechanisms (Hommel et al., 2004; Mayr et al., 2003). In the Simon paradigm, however, these factors are inherently confounded with congruency transitions, precluding definitive adjudication. Nevertheless, the present data provide some traction. Compatibility effects persisted on complete feature alternation trials for both effectors, indicating that spatial interference can arise independently of event-file repetition — arguing against a pure feature-repetition account. Conversely, the effector-specific partial repetition cost argues against a pure conflict-monitoring explanation, pointing to differential binding-related contributions across motor systems. Together, these findings are more consistent with a hybrid account in which top-down control and bottom-up feature-binding operate in parallel — though future work orthogonalizing these factors will be needed to quantify their relative contributions (Schmidt & Weissman, 2014).

### Congruency sequence effect: errors

The pattern of sequential modulation in error rates differed markedly between effectors, as indicated by a significant four-way interaction among stimulus location, response side, previous-trial congruency, and effector. For finger responses, all four Simon contrasts were significant following congruent trials (right response-driven: 14.34%; left response-driven: 8.33%; right stimulus-driven: 13.47%; left stimulus-driven: 9.21%), but were eliminated and in three cases reversed in sign following incongruent trials (all *p*s = 1.00), indicating strong trial-by-trial modulation of spatially driven errors consistent with activation–suppression accounts of conflict adaptation (Ridderinkhof, 2002; Botvinick et al., 2001). For leg responses, no Simon contrasts reached significance in either post-congruent or post-incongruent trials. However, the absence of error-based sequential modulation for legs warrants caution: given near-zero leg error rates (M = 0.41%), a floor effect cannot be ruled out, and the null result may reflect insufficient error variance rather than a genuine absence of accuracy-level conflict adaptation. This contrasts with the RT data, where both effectors showed significant sequential modulation, suggesting that conflict adaptation generalises across effector systems when indexed by response speed, but its expression in accuracy may depend on the availability of sufficient error variance.

### Simon effect across blocks

The within-subjects design, which included congruent and incongruent trials across two effectors within the same session, provides a controlled basis for examining practice effects. Because the Simon effect is a difference score, reductions in its magnitude reflect changes in spatial interference rather than general speed differences affecting both trial types equally. A significant four-way interaction among stimulus position, response side, block, and effector indicates that practice-related changes in spatial conflict resolution differed between effectors, revealing distinct temporal dynamics rather than a uniform reduction across motor systems.

For finger responses, interference remained stable across practice, with minimal change in proportional interference across blocks, consistent with performance near asymptotic efficiency. Given extensive overlearning of fine motor actions, spatial conflict in finger responses is likely already highly optimized, leaving limited scope for short-term improvement (Botvinick et al., 2001; Christiansen & Chater, 2016; Volz et al., 2015). In contrast, leg responses showed a monotonic reduction across practice. All four contrasts declined from 119.7 to 142.4 ms in Block 1 to 71.8–74.6 ms in Block 3, with proportional interference decreasing from 16.45% to 13.61% and 8.78%. This uniform reduction suggests global attenuation of leg-related spatial interference rather than selective modulation of specific components.

By Block 3, proportional interference for legs fell below that of fingers (8.78% vs. 10.76%), producing a crossover that is difficult to reconcile with regression to the mean, which predicts convergence toward a shared baseline rather than a reversal of the initial ordering between effectors. This crossover is instead consistent with practice-related attenuation, potentially reflecting progressive recruitment of top-down control mechanisms that increasingly suppress automatic spatial activation across blocks (Friedman & Robbins, 2022), although this interpretation requires neural confirmation. Future work using delayed retention tests or cross-effector transfer designs will be needed to determine whether this reduction reflects durable learning or transient within-session adaptation.

The observed practice-related divergence across effectors suggests that spatial conflict resolution is not fixed but dynamically shaped by task experience and motor context. Whereas finger responses showed stable interference consistent with near-asymptotic efficiency, leg responses exhibited substantial within-session reductions, indicating greater plasticity in less habitual effector mappings. This pattern extends conflict-adaptation accounts by demonstrating that practice-related optimization is effector-dependent rather than uniform across the motor system. Practice therefore appears to recalibrate the balance between automatic spatial activation and controlled response selection, with greater adaptive gains in lower-limb responding. These findings raise the possibility that leg-based spatial conflict paradigms could provide a useful framework for cognitive–motor training and assessment, with potential relevance for rehabilitation settings—such as gait retraining after stroke or fall-prevention interventions in older adults—where improving lower-limb control under conditions of spatial conflict may be clinically beneficial.

### Theoretical models

The present findings extend existing theoretical accounts of spatial conflict resolution, in which the responding effector has largely been treated as secondary or incidental. The Dual-Route Model (Kornblum et al., 1990; Proctor & Vu, 2006), proposes that Simon task performance arises from the parallel activation of an automatic response pathway driven by spatial location and a controlled pathway guided by task-relevant features. While this framework accounts for the emergence of spatial interference and its modulation by cognitive control, it does not explicitly incorporate effector-dependent parameters within the automatic activation pathway. These findings — the asymmetric distribution of interference across the four contrasts for finger responses, the more symmetric pattern for legs, and the divergent practice-related trajectories across effectors — are consistent with the interpretation that effector type influences not only the overall magnitude but also the internal structure and adaptive dynamics of spatial interference. This suggests that effector-dependent constraints may need to be considered in more comprehensive accounts of Simon task performance. More broadly, these results highlight a limitation of relying exclusively on seated, manual paradigms, which risk treating the manual effector profile as representative of cognitive control more generally.

A motor-constraint account offers an alternative interpretation of these effects. Because leg responses were substantially slower than finger responses, the larger absolute Simon effects for legs may partly reflect the temporal dynamics of automatic spatial activation rather than differences in cognitive control per se. Within diffusion-style accounts of conflict processing — including the Diffusion Model for Conflict tasks (Koob et al., 2023; Luo & Proctor, 2022; Ulrich et al., 2015) — slower response dynamics increase the temporal window over which automatic spatial activation can accumulate prior to response selection, thereby amplifying absolute interference without requiring distinct underlying mechanisms. Consistent with this, proportional interference relative to congruent RT was comparable across effectors (10.6% for fingers, 10.5% for legs), suggesting similar scaling of spatial compatibility effects. However, such proportional equivalence does not preclude differences in the internal structure of interference, which the four-contrast decomposition is well placed to resolve.

The sequential analyses pose a related challenge for the simplest form of conflict-monitoring accounts (Botvinick et al., 2001; Ridderinkhof, 2002), which assume that detecting conflict on one trial triggers a general increase in control that attenuates spatial interference on the next trial, largely irrespective of the specific stimulus-response configuration involved. When previous-trial congruency was represented using the conventional binary congruency variable — the measure such accounts are typically tested against (Gratton et al., 1992) — the resulting sequential effect was small and non-significant for both effectors (finger: M = 1.6 ms, *p* = .786; leg: M = 27.0 ms, *p* = .128). Yet the four-contrast decomposition revealed robust, large sequential modulations for both effectors (finger: M = 54.1 ms, d = 1.356; leg: M = 49.0 ms, d = 0.581), and this adaptation was not distributed uniformly: residual interference after incongruent trials was concentrated in specific contrasts — the right response-driven contrast for fingers, and the left response-driven and left stimulus-driven contrasts for legs — rather than being attenuated equally across all four configurations. A configuration-blind control signal offers no obvious reason why adaptation should fully resolve some stimulus-response pairings while leaving others largely unaffected, or why the affected pairings differ by effector. This pattern instead points toward sequential modulation operating over specific stimulus-response bindings (Hommel et al., 2004; Mayr et al., 2003) and suggests that null or weak CSE reported using collapsed congruency measures may sometimes reflect uneven, configuration-specific adjustments rather than a genuine absence of adaptation.

While still exploratory, this component-level decomposition may also be clinically relevant for populations such as individuals with ADHD, autism spectrum disorder (ASD), schizophrenia, Parkinson’s disease, or stroke, where hemispheric asymmetries, frontostriatal function, and interhemispheric integration are often altered. In neurodevelopmental conditions such as ADHD and ASD, frontostriatal atypicalities and lateralized response-timing deficits, respectively, have been linked to increased susceptibility to interference (D’Cruz et al., 2016; Silk et al., 2016), whereas schizophrenia is associated with altered interhemispheric communication and atypical functional lateralisation during cognitive control (Innocenti et al., 2003). In neurological conditions, stroke-related hemispheric damage can disrupt spatial–motor integration via focal and distributed network effects (Maier et al., 2019), while Parkinson’s disease provides a model of lateralized basal ganglia dysfunction affecting executive control and response selection (Cools et al., 2010). Identifying which specific component of processing is affected could, in principle, provide a more precise characterization of impairment than aggregate congruency scores.

More broadly, the present findings support the Evolutionary Cognitive Enhancement framework, which proposes that cognitive control is rooted in adaptive, movement-based problem-solving systems that are often under-engaged in modern, predominantly sedentary environments (Malaei, 2025; Malaei et al., 2025). Within this framework, effector-specific differences in spatial conflict processing may reflect differences in the recruitment and plasticity of these evolved cognitive–motor systems, with lower-limb engagement potentially reactivating whole-body control mechanisms that are underutilized in conventional hand-based paradigms. This perspective extends beyond purely top-down, rule-based accounts of cognitive control by emphasizing the evolutionary coupling of cognition and action and aligns with embodied theories in which executive functions emerge through continuous interactions among the brain, body, and environment (Raichlen & Alexander, 2017; Zona et al., 2019).

### Methodological considerations

One methodological limitation concerns the confound between effector type and posture. Finger responses were performed while seated and leg responses while standing, such that postural demands and attentional allocation to balance co-varied with effector. This was an intentional design choice motivated by the ecological validity of upright lower-limb action, in which motor control, balance, and spatial cognition are inherently integrated. However, while the Simon effect as a difference score may reduce additive postural influences by controlling for baseline RT differences, posture may also shape the dynamics of response competition itself. Consequently, observed differences between effectors cannot be attributed unambiguously to effector-specific mechanisms and may reflect posture, effector, or their interaction. Future research should independently manipulate posture and effector to disentangle their respective contributions.

Regarding statistical power and trial counts, a post-hoc sensitivity analysis in G*Power (repeated-measures ANOVA, ρ = 0.50, α = 0.05, 1 − β = 0.80) indicated that the study was sufficiently sensitive to detect effect sizes of ηp² ≥ 0.13. This threshold was comfortably exceeded by all primary effects, including the main effect of Effector (ηp² = 0.967), the Stimulus × Response × Effector interaction (ηp² = 0.369), and both CSE interactions (ηp² = 0.655 for fingers; ηp² = 0.258 for legs). In contrast, the four-way interaction (ηp² = 0.003) fell well below this threshold and should therefore be interpreted as preliminary. For the primary Simon effect analyses, previous-trial congruency was derived post hoc rather than experimentally manipulated, yielding approximately 30 trials per stimulus–response cell, which is within the range considered sufficient for stable within-subject RT estimates (Ratcliff, 1993; Whelan, 2008). For sequential analyses, however, splitting trials by previous-trial congruency reduced the effective cell size to approximately 15, which is acknowledged as a limitation. Future work would benefit from increased trial numbers to enable more reliable estimation of finer-grained sequential effects.

Another limitation concerns the sequential variable itself. Previous-trial congruency was coded as a categorical label (congruent vs. incongruent) rather than as a full trial-by-trial specification of stimulus and response features, meaning the analysis cannot disentangle genuine adjustments in top-down control from facilitation or inhibition driven by repetition or alternation of specific features or event-file bindings (Hommel et al., 2004; Mayr et al., 2003). In stimulus–response driven Simon tasks, congruency transitions necessarily co-vary with feature repetition, making it impossible to fully disentangle their independent contributions to sequential effects. Analyses of cleaned trial sequences indicated that transition frequencies were approximately balanced across conditions for both effectors (finger: CC = 26.3%, CI = 24.4%, IC = 25.0%, II = 24.2%, χ²(3) = 3.03, *p* = .387; leg: CC = 25.4%, CI = 25.0%, IC = 25.4%, II = 24.2%, χ²(3) = 1.20, *p* = .752), suggesting that the observed effects are unlikely to reflect systematic transition probability imbalances. Nevertheless, the CSE should be interpreted as reflecting sequential modulation of spatial interference whose mechanistic basis — whether arising from top-down control adjustments, event-file binding, or a combination of both — remains unresolved. Future studies should record full trial-level feature sequences to enable analyses that explicitly control for feature repetition (Schmidt & Weissman, 2014) and to better isolate adaptive control-related contributions from low-level sequence-learning effects.

A direct comparison between the conventional collapsed CSE analysis and the four-contrast decomposition illustrates how analytical choice shapes the characterization of sequential conflict adaptation. When previous-trial congruency is represented using a binary variable that collapses across stimulus location and response side, the resulting CSE is small and non-significant for both finger responses (M = 1.6 ms, *p* = .786) and leg responses (M = 27.0 ms, *p* = .128). In contrast, the four-contrast repeated-measures framework reveals robust sequential modulations for both effectors (finger: M = 54.1 ms, d = 1.356, *p* < .001; leg: M = 49.0 ms, d = 0.581, *p* < .01). By preserving directional stimulus–response configurations and explicitly modelling within-participant variance, this decomposition increases sensitivity to sequential effects operating at the level of specific stimulus–response mappings — effects that are attenuated when heterogeneous conflict transitions are averaged into a single congruency score. This analytical distinction has direct implications for how sequential conflict adaptation is characterised and compared across studies.

### Future directions

Future research should examine how systematic variation in motor engagement shapes cognitive control across healthy and clinical populations, with particular attention to its impact on frontostriatal and sensorimotor network dynamics. A critical next step is to combine the four-contrast decomposition with EEG or fMRI to determine whether effector-specific differences arise during stimulus encoding, response selection, or motor execution — a distinction the present behavioral design cannot adjudicate. It also remains unknown whether embedding cognitively demanding lower-limb actions in predominantly hand-centric environments can reshape functional coupling between motor, parietal, and prefrontal systems, thereby altering the distribution of cognitive–motor control in everyday behavior. At a computational level, extending diffusion-based conflict models to incorporate effector-dependent activation parameters (Koob et al., 2023; Luo & Proctor, 2022; Ulrich et al., 2015) would help clarify whether the observed effector differences reflect context-dependent modulation within a shared control architecture or genuinely distinct instantiations of spatial conflict processing across motor systems.

## Conclusion

By treating stimulus location and response side as independent analytical factors, this study demonstrates that the Simon effect is not a unitary performance index but a composite of distinguishable interference components whose magnitude, internal structure, sequential modulation, and practice-related plasticity are shaped by the responding effector. Although proportional scaling was comparable across effectors — indicating that absolute RT differences primarily reflect baseline speed rather than qualitatively distinct control processes — the four-contrast decomposition revealed divergent internal architectures: a lateralised, response-selection-driven pattern for finger responses and a more bilaterally symmetric organisation for leg responses. Sequential analyses extended previous work by demonstrating robust conflict adaptation in both effectors, including leg-based responses. However, this adaptation was more fully expressed for fingers, whereas leg responses showed residual interference across multiple contrasts, indicating less complete trial-level resolution.

Practice-related analyses revealed strong effector-dependent plasticity. Finger-based interference remained stable across blocks, consistent with asymptotic performance in a highly practiced effector system, whereas leg-based interference declined monotonically with practice, indicating substantial experience-dependent attenuation of spatial compatibility effects. This crossover is inconsistent with regression to the mean and indicative of genuine recalibration of conflict processing in lower-limb control. Together, these findings demonstrate that cognitive control in the Simon task is effector-sensitive: the motor system engaged constrains not only the magnitude of interference but also its internal organisation, its adaptation to recent conflict, and its plasticity over time.

Collectively, these results extend previous accounts of conflict adaptation by showing that sequential effects generalise beyond manual responses to lower-limb actions, while also revealing systematic differences in their expression across effectors. They further identify effector-specific learning dynamics as a neglected dimension of cognitive control, with lower-limb systems showing greater capacity for performance change under practice. This has direct implications for theoretical models that treat the responding effector as incidental, as well as for assessment frameworks that rely predominantly on seated manual paradigms as proxies for executive function. Consistent with embodied and evolutionary perspectives, the findings highlight the role of sensorimotor engagement in shaping cognitive control and suggest that lower-limb paradigms may provide a complementary window into adaptive control mechanisms with potential relevance for cognitive assessment and rehabilitation. Future work should clarify the neural mechanisms underlying these effector-specific effects and disentangle posture, effector, and feature-repetition contributions using fully orthogonalized, trial-level sequence designs capable of isolating adaptive control from low-level learning effects.

## Data Availability

Data are available from the authors upon reasonable request.

## Code availability

Analysis code is available from the authors upon reasonable request.
